# Likelihood of HIV and recent bacterial sexually transmitted infections among transgender and non-binary individuals in 20 European countries, October 2023 to April 2024

**DOI:** 10.2807/1560-7917.ES.2024.29.48.2400347

**Published:** 2024-11-28

**Authors:** Haoyi Wang, Johann Kolstee, Jules L Casalini, Samira Hakim, Hanne ML Zimmermann, Kai J Jonas

**Affiliations:** 1 Department of Work and Social Psychology, Maastricht University, Maastricht, the Netherlands; 2 Transgender Netwerk Nederland, Amsterdam, the Netherlands

**Keywords:** Transgender, non-binary, HIV, bacterial sexually transmitted infections, Europe, STI, surveillance

## Abstract

**Background:**

Global data highlight the disproportionate burden of HIV and sexually transmitted infections (STIs) among transgender individuals. However, scant data exist for both transgender and non-binary individuals in European HIV/STI surveillance.

**Aim:**

To assess self-reported prevalence of HIV and bacterial STIs (syphilis, gonorrhoea, chlamydia) in the past 6 months among transgender and non-binary individuals, comparing the likelihoods of recent STIs between groups.

**Methods:**

Using data from the cross-sectional PROTECT survey conducted in 20 European countries from October 2023 to April 2024, we analysed a subset of 452 participants, 178 transgender and 274 non-binary individuals. Logistic regression was used to compare the risk of each recent bacterial STI, and Poisson regression to compare the risk of the number of recent STIs.

**Results:**

Among transgender individuals, 5 (2.8%) self-reported HIV infection, and recent STI prevalence was 6.7% for syphilis, 15.6% for gonorrhoea and 19.6% for chlamydia. For non-binary individuals, 15 (5.5%) self-reported HIV infection and recent STI prevalence was 15.0% for syphilis, 18.7% for gonorrhoea and 20.8% for chlamydia. Non-binary individuals had significantly higher risk for syphilis (aOR: 1.81; 95% CI: 1.01–4.05) and multiple recent STIs (aOR: 1.46; 95% CI: 1.11–1.91) compared with transgender individuals.

**Conclusion:**

While both transgender and non-binary individuals showed high self-reported prevalence of HIV and bacterial STIs, non-binary individuals showed greater prevalence of STIs, particularly syphilis. Efforts aimed at HIV/STI prevention and surveillance should encourage inclusion of those who identify as non-binary and other gender-diverse individuals alongside transgender individuals to enhance the provision of tailored prevention and treatment services in Europe.

**Key public health message**

**What did you want to address in this study and why?**

There remains a notable gap in understanding the prevalence of sexually transmitted infections (STI) among both transgender and non-binary individuals in Europe and HIV/STI surveillance and research. We collected sociodemographic and behavioural data from transgender and non-binary individuals to understand the likelihood of self-reported HIV infection and other STIs among these groups in 20 European countries.

**What have we learnt from this study?**

We learnt that transgender and non-binary individuals in Europe have a high likelihood of HIV infection and bacterial STIs (syphilis and gonorrhoea). Non-binary individuals reported higher likelihood for syphilis (twofold) and having more STIs (1.5-fold) vs transgender individuals. Also, one in five individuals had never tested for HIV, around 15% had never tested for STIs and the majority (70%) had never used HIV pre-exposure prophylaxis (PrEP).

**What are the implications of your findings for public health?**

Our findings underscore substantial unmet health needs among transgender and non-binary individuals in Europe. Inclusivity for transgender, non-binary or other gender-diverse populations in HIV/STI research and surveillance in Europe is urgently needed to ensure that the distinct needs of key populations are adequately addressed, and adequate measures are being put into place.

## Introduction

For more than a decade, transgender and non-binary individuals have increasingly shifted into the sexual healthcare focus [1-3], although there is a notable lack of research and surveillance data on these populations, including HIV and sexually transmitted infections (STIs) [4]. The term ‘trans*’ is often used as an umbrella term for individuals whose gender identity does not align with the sex they were assigned at birth. This term includes binary transgender men and women (referred to in this study as ‘transgender’ individuals), as well as non-binary and other gender-diverse individuals. Non-binary individuals explicitly reject binary gender constructions and identify with a wide range of gender expressions, distinct from binary transgender identities [1].

While there are more data available on HIV and STIs among transgender individuals, little is known about the risk for HIV infection and STIs among non-binary people. Global meta-analytical evidence indicates that transgender individuals are disproportionately affected by HIV infection [2,5], with transgender women facing a 66 times higher risk of infection compared with cisgender men, and transgender men facing a risk 6.8 times higher [2]. In addition, other STIs often interact syndemically with HIV infections [2,6-9]. Both transgender men and women are also at increased risk for bacterial STIs, such as syphilis, gonorrhoea and chlamydia [10], with a meta-analysis synthesising self-reported history of STI of 22% in the United States (US) context [11]. However, such US data-based synthesis may not be applicable to a European context, because of the different ethnic composition of the population and different migrant countries of origin. Research focusing specifically on HIV and STI prevalence or incidence among non-binary individuals in Europe is sparse, with a few exceptions like studies in the United Kingdom (UK) [12,13], Germany [14], or ongoing research projects for which data are not yet available [15]. The lack of data is mainly attributed to challenges in recruiting adequate sample sizes of non-binary individuals, difficulties obtaining representative samples and issues with community trust [16].

The gaps in European surveillance data for transgender and non-binary individuals are concerning, and can hamper public health interventions. While countries like France, the Netherlands and Ireland have taken steps to include transgender data in their national HIV surveillance, information about the HIV prevalence among transgender individuals in many other European countries, as well as data on non-binary individuals, remains scarce [17]. Similarly, within European STI surveillance, there is a notable absence of data for both groups [18], stemming from inadequate and non-inclusive data collection methods. For example, uniformity is lacking across countries with regards to data collection and the reporting of STI and HIV prevalence in these groups [18]. In addition, the misclassification of their identities (such as categorising these groups as men who have sex with men (MSM) [16]), or outright exclusion of their data also still occurs [17-19]. Nevertheless, surveillance data gaps prevent analysis of risk and understanding/assessment of variation between transgender and non-binary individuals.

Given that HIV infection and STI risk among transgender and non-binary individuals can be a dynamic phenomenon, e.g. seasonal or level of transition specific [2], it is important to understand the individual risk factors that could determine a higher risk of HIV infection and STIs among these groups. Previous studies have identified several primary risk factors for an HIV and STI infection, including younger age [20], engaging in high-risk sexual behaviours such as condomless intercourse and receiving or providing transactional sex [21-23], substance use or chemsex [20,22-24]. However, these findings are primarily based on research conducted among transgender individuals, overlooking the unique experiences of non-binary people. Furthermore, these studies were not performed within a European context, indicating a notable knowledge gap specific to Europe. Therefore, there's a need for research that specifically focuses on both transgender and non-binary populations to update our understanding of the risk factors and potential differences in these risk factors for HIV and STIs within these populations from a European perspective.

To better bridge the current data gaps on the likelihood of HIV and other STIs among transgender and non-binary populations in Europe, we assessed the self-reported prevalence of HIV, and recent bacterial STIs (syphilis, gonorrhoea and chlamydia) in the preceding 6 months among transgender and non-binary individuals in a pan-European context. We also compared the risks of acquiring STIs between these two groups. Additionally, our investigation delved into identifying the determinants contributing to recent bacterial STIs among transgender and non-binary individuals.

## Methods

### Study context and population

We conducted a cross-sectional online survey in 20 European countries (Austria, Belgium, Cyprus, Czechia, Denmark, Finland, France, Germany, Greece, Ireland, Italy, Luxemburg, the Netherlands, Norway, Poland, Portugal, Spain, Sweden, Switzerland and the UK) from October 2023 to April 2024, named PROTECT [25]. In brief, the survey was distributed via social media platforms (e.g. Instagram), gay dating apps (e.g. Grindr) and the survey website (https://protect-study.eu) in 22 languages, covering mainly European languages and some key non-European migrant languages [26]. The PROTECT survey sought to understand the extent to which European MSM, trans* and heterosexual individuals are interested and intend to use long-acting pre-exposure prophylaxis (LA-PrEP) when made available. In addition, people with HIV (PWH) were also included to explore their attitudes towards their HIV-negative partner’s use of PrEP modalities.

In the present analysis, we included only subsamples from transgender and non-binary individuals. The full study procedure, full survey and recruitment have been described elsewhere [26]. 

### Definitions

Participants in PROTECT were defined for this study following prior consultation with trans* community members and the previous recommendations by Schudson and Morgenroth [27]. We defined binary transgender individuals as those who self-reported their binary gender identities as transgender men or transgender women, and those whose self-reported binary gender mismatched their self-reported sex assigned at birth (e.g. identifying as ‘woman’ but having reported sex assigned at birth as ‘male’). We further defined non-binary individuals as those who did not report their gender as either ‘man’ or ‘woman’. This includes participants who explicitly reported a ‘non-binary’ identity as well as those who reported other gender experiences (e.g. ‘other gender’ or ‘prefer not to disclose’).

### Variables

All outcome variables were self-reported, including HIV infection status (‘positive’, ‘negative’ or ‘unaware’), recent syphilis diagnosis in the preceding 6 months (‘yes’ or ‘no’), recent gonorrhoea diagnosis in the preceding 6 months (‘yes’ or ‘no’) and recent chlamydia diagnosis in the preceding 6 months (‘yes’ or ‘no’). Furthermore, the types of self-reported bacterial STIs in the preceding 6 months were counted from 0 to 3, where 0 indicates no bacterial STI was diagnosed in the preceding 6 months and 3 indicates all three types of the bacterial STI (syphilis, gonorrhoea and chlamydia) were diagnosed in the preceding 6 months.

Sociodemographic variables included age, education attainment, employment status, perceived financial status, place of residence and migration background. Behavioural variables included relationship status, condomless anal intercourse (CAI), CAI by PWH, CAI while not on PrEP or using PrEP but with suboptimal adherence (unprotected sex), the number of sexual partners, transactional sex (receiving sex) or transactional sex (providing sex), chemsex in the preceding 6 months, HIV and STI testing frequencies and oral PrEP use status. 

### Statistical analysis

We used descriptive statistical analyses to define our samples in terms of sociodemographic, behavioural and outcome variables between the transgender and non-binary groups. A chi-square test was performed to compare the differences in each variable between these two groups.

Next, given that both HIV status and oral PrEP use status were our key variables of interest in this study, and that oral PrEP was only available to HIV-negative individuals, we conducted a stratified analysis based on participants’ HIV status. Firstly, among all participants who had ever tested for STIs, we performed univariable logistic regression analyses to examine the associations between HIV status and recent diagnoses of each of the three STIs. Secondly, for HIV-negative participants who ever tested for STIs, we conducted multivariable logistic regression analyses to compare the likelihood of each recent STI between transgender and non-binary individuals. We performed univariable logistic regression modelling with each sociodemographic and behavioural determinant to investigate potential correlations with having a recent diagnosis of each STI. We conducted stepwise selection by assessing the model fits based on the lowest value of the Akaike’s Information Criterion (AIC). We retained participants’ gender identity in the models, regardless of its statistical significance, given its importance as primary variable of interest. Thirdly, we applied this approach separately to transgender and non-binary individuals to explore potential variations in the determinants of recent STI diagnoses across these groups. Models’ variance inflation factors (VIFs) were calculated to assess the potential multi-collinearity and confounders. Finally, given the counted nature of the measurement of the number of types of STI, we conducted a univariable Poisson regression analysis to compare the number of recent STIs in the past 6 months among transgender and non-binary individuals.

Regression diagnostics for all regression analyses did not reveal collinearity, conspicuous values or normality violations. P values of < 0.05 were considered significant. All analyses were conducted in R (version 4.3.2).

## Results

**Figure 1:**
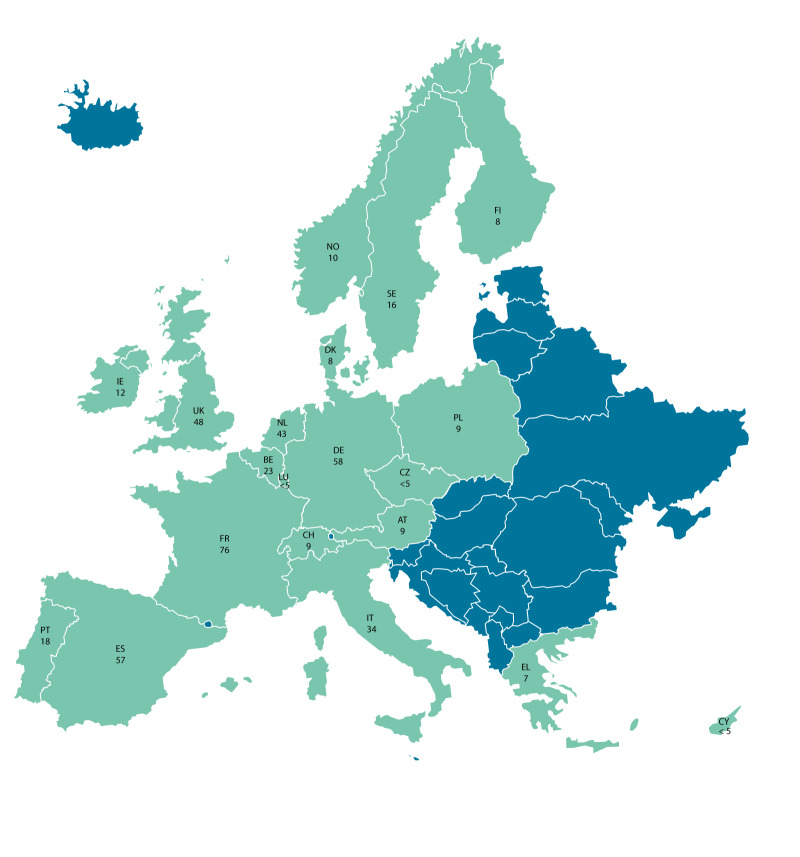
Country of residence of transgender and non-binary participants from the PROTECT Study in 20 European countries, October 2023–April 2024 (n = 452)

AT: Austria; BE: Belgium; CH: Switzerland; CY: Cyprus; CZ: Czechia; DE: Germany; DK: Denmark; ES: Spain; FI: Finland; FR: France; EL: Greece; IE: Ireland; IT: Italy; LU: Luxembourg; NL: Netherlands; NO: Norway; PL: Poland; PT: Portugal; SE: Sweden; UK: United Kingdom.

Of the 15,458 samples collected from the PROTECT survey, 452 individuals self-reported as either transgender (n = 178; 39%) or non-binary (n = 274; 61%) and were included in this analysis. Of the transgender individuals, 63 (35%) self-identified as transgender women and 115 (65%) self-identified as transgender men. Of the non-binary individuals, 213 (78%) reported a non-binary identity, 35 (13%) reported other identities and 26 (10%) preferred not to disclose. The majority of the trans* individuals in our study were living in European countries with larger population sizes, such as France, Germany, Spain, the UK and Italy (Figure 1).

For countries where the sample size was smaller than 5, we did not present the actual number but marked with ‘< 5’. Green: Countries where the PROTECT survey was conducted. Blue: Countries where the PROTECT survey was not conducted.

Overall, the median age was 30 years (interquartile range (IQR): 25–39); transgender individuals had a median age of 30 years (IQR: 25–38) and for non-binary individuals, the median age was 31 years (IQR: 25–40). Compared with transgender individuals, non-binary individuals were less likely to be financially advantaged (p = 0.019), more likely, yet statistically insignificant, to pay for transactional sex (p = 0.064) but less likely to receive payment for transactional sex (p < 0.001). No major differences were found in the migrant status and country of origin (results not shown) between transgender and non-binary samples. Table 1 summarises other sociodemographic and behavioural characteristics.

**Table 1:** Sociodemographic and behavioural characteristics of the transgender and non-binary study participants in 20 European countries, October 2023–April 2024 (n = 452)

Determinant		Transgender individuals (n = 178)		Non-binary individuals (n = 274)		Total sample (n = 452)		p value^a^
		n	%	n	%	n	%	
Sociodemographic characteristics								
Age (years)	Median (IQR)	30	25–38	31	25–40	30	25–39	0.876
	18–24	40	22.5	59	21.5	99	21.9	0.450
	25–29	47	26.4	64	23.4	111	24.6	
	30–39	52	29.2	80	29.2	132	29.2	
	40–49	25	14.0	40	14.6	65	14.4	
	50–59	9	5.1	17	6.2	26	5.8	
	60–69	2	1.1	8	2.9	10	2.2	
	≥ 70	3	1.7	6	2.2	9	2.0	
Education attainment	Less than secondary education	13	7.3	13	4.7	26	5.8	0.154
	Secondary education (high school or equivalent)	73	41.0	90	32.8	163	36.1	
	Bachelor’s degree (university or equivalent)	50	28.1	84	30.7	134	29.6	
	Master’s degree (university or equivalent)	36	20.2	69	25.2	105	23.2	
	PhD/doctorate	6	3.4	18	6.6	24	5.3	
Employment status	Employed	88	49.4	136	49.6	224	49.6	0.077
	Other	25	14.0	25	9.1	50	11.1	
	Retired or on medical leave	17	9.6	17	6.2	34	7.5	
	Student	24	13.5	61	22.3	85	18.8	
	Unemployed	24	13.5	35	12.8	59	13.1	
Perceived financial status	Living really comfortably on present income	2	1.1	7	2.6	9	2.0	0.019
	Living comfortably on present income	34	19.1	40	14.6	74	16.4	
	Neither comfortable nor struggling on present income	59	33.1	112	40.9	171	37.8	
	Struggling on present income	37	20.8	69	25.2	106	23.5	
	Really struggling on present income	46	25.8	46	16.8	92	20.4	
Place of residence	Very big city (one million or more people)	43	24.2	81	29.6	124	27.4	0.628
	Big city (500,000–999,999 people)	34	19.1	42	15.3	76	16.8	
	Medium-sized city (100,000–499,999 people)	49	27.5	72	26.3	121	26.8	
	Small city (10,000–99,999 people)	34	19.1	47	17.2	81	17.9	
	Village/countryside (less than 10,000 people)	18	10.1	32	11.7	50	11.1	
Migration background	Non-migrant	108	60.7	169	61.7	277	61.1	0.410
	First generation migrant	47	26.4	80	29.2	127	28.1	
	Second generation migrant	23	12.9	25	9.1	48	10.6	
Behavioural characteristics								
Relationship status	Single	38	21.3	92	33.6	130	28.8	0.021
	Dating	66	37.1	65	23.7	131	29.0	
	In a monogamous relationship	35	19.7	50	18.2	85	18.8	
	In an open/polyamorous relationship	39	21.9	67	24.5	106	23.5	
Condomless anal intercourse in the preceding 6 months	No	32	18.0	42	15.3	74	16.4	0.153
	Yes	146	82.0	232	84.7	378	83.6	
Condomless anal intercourse with people living with HIV in the preceding 6 months	No	178	100.0	270	98.5	448	99.1	0.386
	Yes	0	0.0	4	1.5	4	0.9	
Unprotected sex in the preceding 6 months^b^	Yes	124	69.7	191	69.7	315	69.7	0.565
	No	54	30.3	83	30.3	137	30.3	
Number of sexual partners in the preceding 6 months	0	11	6.2	27	9.9	38	8.4	0.315
	1	31	17.4	46	16.8	77	17.0	
	2–10	68	38.2	87	31.8	155	34.3	
	11–50	38	21.3	65	23.7	103	22.8	
	51–100	4	2.2	15	5.5	19	4.2	
	101–150	5	2.8	4	1.5	9	2.0	
	≥ 150	21	11.8	30	10.9	51	11.3	
Transactional sex (receiving) in the preceding 6 months	No	160	89.9	228	83.2	388	85.8	0.064
	Yes	18	10.1	46	16.8	64	14.2	
Transactional sex (providing) in the preceding 6 months	No	114	64.0	195	71.2	309	68.4	< 0.001
	Yes	64	36.0	79	28.8	143	31.6	
Chemsex in the preceding 6 months^c^	No	156	87.6	235	85.8	391	86.5	0.668
	Yes	22	12.4	39	14.2	61	13.5	
HIV testing frequency	Frequently testing	56	31.5	96	35.0	152	33.6	0.319
	Every 6 months	32	18.0	60	21.9	92	20.4	
	Once per year	34	19.1	37	13.5	71	15.7	
	Less than once per year	31	17.4	13	4.7	44	9.7	
	Never	25	14.0	68	24.8	93	20.6	
STI testing frequency	Frequently testing	56	31.5	82	29.9	138	30.5	0.583
	Every 6 months	32	18.0	57	20.8	89	19.7	
	Once per year	34	19.1	46	16.8	77	17.0	
	Less than once per year	31	17.4	40	14.6	74	16.4	
	Never	25	14.0	49	17.9	74	16.4	
Oral PrEP use status	Current	38	21.3	72	26.3	110	24.3	0.170
	Former	10	5.6	22	8.0	32	7.1	
	Naive	125	70.2	165	60.2	290	64.2	
	NA^d^	5	2.8	15	5.5	20	4.4	

IQR: interquartile range; NA: not applicable; PrEP: pre-exposure prophylaxis; STI: sexually transmitted infection.

^a ^A chi-square test was applied to test the differences between transgender and non-binary groups.

^b^ Unprotected sex was defined as condomless intercourse without using HIV pre-exposure prophylaxis (PrEP) or with suboptimal PrEP adherence.

^c^ Use of stimulant drugs (ecstasy/MDMA, cocaine, amphetamine, crystal methamphetamine, mephedrone or ketamine) to make sexual experiences more intense or last longer.

^d^ Not applicable because of living with HIV.

### Self-reported prevalence of HIV and recent bacterial STIs

**Figure 2:**
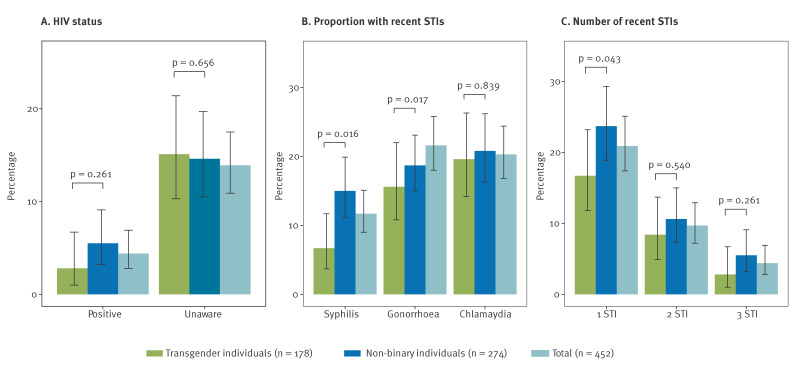
Prevalence of self-reported HIV status and recent STI diagnoses among transgender and non-binary individuals in 20 European countries, October 2023–April 2024 (n = 452)

Figure 2 summarises self-reported HIV prevalence, recent STIs (syphilis, gonorrhoea and chlamydia) and cumulative types of recent STIs (from 0 to all 3) among transgender and non-binary individuals, for the detailed numbers, see Supplementary Table S1. Across our overall sample, 4.4% (95% CI: 2.8–6.9) of participants reported living with HIV, 81.7% (95% CI: 77.7–85.1) had a negative status, and 13.9% (95% CI: 10.9–17.5) were unaware of their status. Recent syphilis, gonorrhoea and chlamydia diagnoses were reported by 11.7% (95% CI: 9.0–15.1), 21.6% (95% CI: 18.0–25.8) and 20.3% (95% CI: 16.8–25.4), respectively. Additionally, 20.9% (95% CI: 17.4–25.1), 9.7% (95% CI: 7.2–12.9) and 4.4% (95% CI: 2.8–6.9) of our overall samples reported one, two or all three types of recent bacterial STIs in the preceding 6 months.

STI: sexually transmitted infection.

Recent STIs indicate self-reported STIs in the preceding 6 months. Differences between transgender and non-binary individuals were tested by a chi-square test. Error bars show 95% confidence intervals using continuity correction methods. Detailed information, including the n numbers, can be found in Supplementary Table S1.

Among transgender individuals, 2.8% (95% CI: 1.0–6.7) reported living with HIV, 82.1% (95% CI: 75.5–87.3%) had a negative status and 15.1% (95% CI: 10.3–21.4) were unaware of their status. Recent syphilis, gonorrhoea and chlamydia diagnoses were reported by 6.7% (95% CI: 3.7–11.7), 15.6% (95% CI: 10.8–22.0) and 19.6% (95% CI: 14.2–26.3%) respectively, with 16.7% (95% CI: 11.8–23.2), 9.4% (95% CI: 4.9–13.7) and 2.8% (95% CI: 1.0–6.7) reporting one, two or all three types of bacterial STIs. Non-binary individuals had a higher, but non-significant, self-reported HIV prevalence (5.5%; 95% CI: 3.2–9.1%, p = 0.261), but lower reports of being unaware of HIV status (14.6%; 95% CI: 10.5–19.7%, p = 0.656). They also reported higher recent STI diagnoses (significantly higher syphilis: 15.0%; 95% CI: 11.1–19.9%, p = 0.016; significantly higher gonorrhoea: 18.7%; 95% CI: 15.0–23.1%, p = 0.017; and similar chlamydia: 20.8%; 95% CI: 16.3–26.2%, p = 0.839) and multiple recent STI diagnoses (one STI: 23.7%; 95% CI: 18.9–29.3%, p = 0.043; two STIs: 10.6%; 95% CI: 7.3–15.0%, p = 0.540; three STIs: 5.5%; 95% CI: 3.2–9.1%, p = 0.261) compared with transgender individuals.

### Likelihood of recent STIs between transgender and non-binary individuals

Among the total samples, univariably, both recent syphilis and gonorrhoea diagnoses were positively associated with a positive HIV status (OR: 8.71; 95% CI: 3.37–22.57 and OR: 2.86; 95% CI: 1.12–7.15, respectively). A recent chlamydia diagnosis was not significantly associated with a positive HIV status (OR: 1.55; 95% CI: 0.53–3.99). The full univariable logistic regression analysis on the likelihood of recent self-reported STIs is provided in Supplementary Table S2. Table 2 outlines the detailed information from the multivariable logistic regression analyses that compared the risk of each recent STI between the HIV-negative transgender and non-binary individuals who ever tested for STIs. Compared with transgender individuals, non-binary individuals showed a significantly higher likelihood of recent syphilis infections (aOR: 1.81; 95% CI: 1.01–4.05).

**Table 2:** Multivariable logistic regression on the likelihoods of recent self-reported STI diagnosis among HIV-negative transgender and non-binary individuals who ever tested for STIs in 20 European countries, October 2023–April 2024 (n = 369)

Determinant		Recent syphilis diagnosis^a^			Recent gonorrhoea diagnosis^b^			Recent chlamydia diagnosis^c^		
		aOR	95% CI	p value	aOR	95% CI	p value	aOR	95% CI	p value
Sociodemographic characteristics										
Gender	Transgender	Ref.			Ref.			Ref.		
	Non-binary	1.81	1.01–4.05	0.047	1.42	0.79–2.53	0.235	0.84	0.49–1.45	0.538
Education attainment	Less than secondary education	NA			Ref.			NA		
	Secondary education (high school or equivalent)				0.18	0.05–0.64	0.008			
	Bachelor’s degree (university or equivalent)				0.42	0.12–1.45	0.475			
	Master’s degree (university or equivalent)				0.38	0.11–1.34	0.656			
	PhD/doctorate				0.49	0.09–2.43	0.982			
Place of residence	Very big city (one million or more people)	NA			Ref.			NA		
	Big city (500,000–999,999 people)				0.70	0.31–1.54	0.374			
	Medium-sized city (100,000–499,999 people)				0.43	0.21–0.91	0.025			
	Small city (10,000–99,999 people)				0.60	0.25–1.40	0.238			
	Village/countryside (less than 10,000 people)				0.17	0.04–0.71	0.014			
Behavioural characteristics										
Transactional sex (receiving) in the preceding 6 months	No	Ref.			NA			NA		
	Yes	4.15	1.81–9.48	< 0.001						
Chemsex in the preceding 6 months	No	Ref.			NA			Ref.		
	Yes	3.79	1.68–8.57	< 0.001				2.23	1.13–4.37	0.019
HIV testing frequency	Frequently testing	Ref.			Ref.			Ref.		
	Every 6 months	2.42	0.94–6.21	0.066	2.42	1.13–5.20	0.023	1.36	0.68–2.72	0.382
	Once per year	1.60	0.45–5.70	0.466	0.64	0.18–2.31	0.498	0.81	0.33–2.03	0.661
	Less than once per year	1.06	0.18–6.17	0.951	0.83	0.31–2.23	0.706	0.54	0.16–1.87	0.338
	Never	0.00	0.00–Inf.	0.998	0.00	0.00–Inf.	0.997	0.06	0.01–0.52	0.010
Oral PrEP use status	Current	Ref.			Ref.			Ref.		
	Former	0.82	0.28–2.22	0.704	0.55	0.21–1.44	0.225	0.57	0.23–1.40	0.408
	Naive	0.16	0.07–0.36	< 0.001	0.20	0.09–0.44	< 0.001	0.26	0.12–0.52	< 0.001

aOR: adjusted odds ratio; CI: confidence intervals; Inf.: infinity; NA: not applicable; PrEP: pre-exposure prophylaxis; Ref.: reference.

Univariable regression results can be found in Supplementary Table S2.

^a ^The variance inflation factors for these variables ranged from 1.04 to 2.07, suggesting negligible multi-collinearity.

^b ^The variance inflation factors for these variables ranged from 1.05 to 2.01, suggesting negligible multi-collinearity.

^c ^The variance inflation factors for these variables ranged from 1.05 to 1.91, suggesting negligible multi-collinearity.

### Determinants of recent STIs among and between transgender and non-binary individuals

Table 2 summarises the determinants of recent STIs among both HIV-negative transgender and non-binary individuals. Recent syphilis diagnosis was positively associated with receiving transactional sex (aOR: 4.15; 95% CI: 1.81–9.48) and engaging in chemsex (aOR: 3.79; 95% CI:  1.68–8.57) in the preceding 6 months. Conversely, being PrEP naive (aOR: 0.16; 95% CI: 0.07–0.36) decreased the likelihood of a recent syphilis diagnosis. For gonorrhoea, testing HIV every 6 months (aOR: 2.42; 95% CI: 1.13–5.20) was associated with recent diagnosis, while having secondary education (aOR: 0.18; 95% CI: 0.05–0.64) and being PrEP naive (aOR: 0.20; 95% CI: 0.09–0.44) decreased the likelihood. For chlamydia, never tested for HIV (aOR: 0.06; 95% CI: 0.01–0.52) and being PrEP naive (aOR: 0.26; 95% CI: 0.12–0.52) decreased the likelihood of recent chlamydia diagnosis.

There are notable differences in the determinants of recent STIs between transgender and non-binary individuals as outlined in Table 3. The full univariable logistic regression analysis on the likelihood of recent self-reported STIs among HIV-negative transgender individuals is provided in Supplementary Table S3, and among HIV-negative non-binary individuals is provided in Supplementary Table S4. Univariably, for recent syphilis, living with HIV was significantly associated with recent diagnoses among both transgender and non-binary individuals (OR: 10.15; 95% CI: 1.22–69.35 and OR: 7.65; 95% CI: 2.56–23.37, respectively). For recent gonorrhoea, living with HIV showed no significant association with a recent diagnosis among transgender individuals (OR: 1.27; 95% CI: 0.06–9.07). However, it was significantly associated with recent gonorrhoea diagnoses among non-binary individuals (OR: 3.25; 95% CI: 1.12–9.66). For recent chlamydia, living with HIV showed no significant association with a recent diagnosis among both transgender and non-binary individuals (OR: 0.89; 95% CI: 0.04–6.29 and OR: 1.82; 95% CI: 0.55–5.39, respectively).

**Table 3:** Multivariable logistic regression on the determinant of recent self-reported STI diagnosis among HIV-negative transgender and non-binary individuals in 20 European countries, October 2023–April 2024 (n = 369)

Determinant		Recent syphilis diagnosis						Recent gonorrhoea diagnosis						Recent chlamydia diagnosis					
		Transgender individuals^a^			Non-binary individuals^b^			Transgender individuals^c^			Non-binary individuals^d^			Transgender individuals^e^			Non-binary individuals^f^		
		aOR	95% CI	p value	aOR	95% CI	p value	aOR	95% CI	p value	aOR	95% CI	p value	aOR	95% CI	p value	aOR	95% CI	p value
Migrant background	Non-migrant	Ref.			NA			NA			NA			NA			NA		
	First generation migrant	7.97	1.80–35.18	0.006															
	Second generation migrant	1.43	0.12–16.80	0.776															
Education attainment	Less than secondary education	NA			NA			NA			Ref.			NA			NA		
	Secondary education (high school or equivalent)										0.10	0.02–0.55	0.009						
	Bachelor’s degree (university or equivalent)										0.32	0.06–1.67	0.176						
	Master’s degree (university or equivalent)										0.24	0.04–1.28	0.094						
	PhD/doctorate										0.30	0.04–2.24	0.241						
Place of residence	Very big city (one million or more people)	NA			NA			NA			NA			NA			Ref.		
	Big city (500,000–999,999 people)																0.70	0.37–1.36	0.307
	Medium-sized city (100,000–499,999 people)																0.36	0.27–1.90	0.537
	Small city (10,000–99,999 people)																0.32	0.15–0.91	0.034
	Village/countryside (less than 10,000 people)																0.09	0.12–1.02	0.067
Transactional sex (receiving) in the preceding 6 months	No	Ref.			Ref.			NA			NA			NA			Ref.		
	Yes	4.44	1.00–19.67	0.049	4.45	1.88–10.62	< 0.001										3.07	1.31–7.18	0.009
Chemsex in the preceding 6 months	No	Ref.			Ref.			NA			NA			Ref.			Ref.		
	Yes	5.46	1.09–27.38	0.039	4.28	1.76–10.50	0.001							3.09	0.93–10.29	0.066	2.14	0.87–5.22	0.092
HIV testing frequency	Frequently testing	NA			NA			Ref.			Ref.			NA			NA		
	Every 6 months							1.02	0.37–2.64	0.995	2.28	0.93–5.65	0.073						
	Once per year							0.21	0.03–0.85	0.043	8.90	0.27–2.92	0.848						
	Less than once per year							0.25	0.04–1.02	0.087	0.75	0.13–4.22	0.748						
	Never							0.00	0.00–Inf.	0.987	0.00	0.00–Inf.	0.997						
Oral PrEP use status	Current	Ref.			Ref.			Ref.			Ref.			Ref.			Ref.		
	Former	2.30	0.25–17.50	0.426	0.51	0.14–1.62	0.276	0.84	0.13–4.69	0.851	0.55	0.18–1.73	0.307	0.42	0.09–1.98	0.277	0.95	0.31–2.75	0.919
	Naive	0.18	0.03–0.92	0.041	0.12	0.04–0.30	< 0.001	0.44	0.10–1.77	0.256	0.15	0.06–0.39	< 0.001	0.15	0.06–0.37	< 0.001	0.21	0.10–0.44	< 0.001

aOR: adjusted odds ratio; CI: confidence intervals; Inf.: infinity; NA: not applicable; PrEP: pre-exposure prophylaxis, Ref.: reference.

Univariable regression results can be found in Supplementary Table S3–4.

^a^ The variance inflation factors for these variables ranged from 1.04 to 1.24, suggesting negligible multi-collinearity.

^b^ The variance inflation factors for these variables ranged from 1.09 to 1.27, suggesting negligible multi-collinearity.

^c^ The variance inflation factors for these variables ranged from 1.30 to 2.58, suggesting negligible multi-collinearity.

^d^ The variance inflation factors for these variables ranged from 1.13 to 1.86, suggesting negligible multi-collinearity.

^e^ The variance inflation factors for these variables ranged from 1.18 to 2.64, suggesting negligible multi-collinearity.

^f^ The variance inflation factors for these variables ranged from 1.11 to 1.24, suggesting negligible multi-collinearity.

For transgender individuals, multivariably, being a first-generation migrant (aOR: 7.97; 95% CI: 1.80–35.18), receiving transactional sex (aOR: 4.44; 95% CI: 1.00–19.67) and engaging in chemsex in the preceding 6 months (aOR: 5.46; 95% CI: 1.09–27.38) increased the likelihood of a recent syphilis diagnosis. Conversely, being PrEP naive (aOR: 0.18; 95% CI: 0.03–0.92) decreased the likelihood of a recent syphilis diagnosis; testing HIV once per year (aOR: 0.21; 95% CI: 0.03–0.85) decreased the likelihood of a recent gonorrhoea diagnosis; and similarly, being PrEP naive (0.15; 95% CI: 0.06–0.37) decreased the likelihood of a recent chlamydia diagnosis.

For non-binary individuals, multivariably, having received transactional sex (aOR: 4.45; 95% CI: 1.88–10.62) and engaging in chemsex (aOR: 4.28; 95% CI: 1.76–10.50) in the preceding 6 months increased the likelihood of a recent syphilis diagnosis. Conversely, being PrEP naive (aOR: 0.12; 95% CI: 0.04–0.30) decreased this likelihood. Additionally, having a secondary education (aOR: 0.10; 95% CI: 0.02–0.55) and being PrEP naive (aOR: 0.15, 0.06–0.39) reduced the likelihood of a recent gonorrhoea diagnosis. Moreover, having received transactional sex (aOR: 3.07; 95% CI: 1.31–7.18) in the preceding 6 months increased the likelihood of a recent chlamydia diagnosis, while living in a small-sized city (aOR: 0.32; 95% CI: 0.15–0.91) and being PrEP naive (aOR: 0.21; 95% CI: 0.10–0.44) decreased its likelihood.

### Risk of multiple recent STIs between transgender and non-binary individuals

Compared with transgender individuals, non-binary individuals demonstrated a significantly higher risk of having multiple recent bacterial STIs (OR: 1.46; 95% CI: 1.11–1.91). Table 4 outlines the detailed information of the Poisson regression model.

**Table 4:** Univariable Poisson regression on the risk of multiple recent STIs among transgender and non-binary individuals in 20 European countries, October 2023–April 2024 (n = 452)

Determinant		Having higher number of the of STI types (0–3)		
		OR	95% CI	p value
Gender group	Transgender	Ref.		
	Non-binary	1.46	1.11–1.91	0.007

CI: confidence interval; OR: odds ratio; STI: sexually transmitted infection.

STIs included syphilis, gonorrhoea and chlamydia.

## Discussion

The findings from 452 transgender and non-binary individuals in 20 European countries showed that both groups face high risks of HIV and the likelihood to acquire recent bacterial STIs. Although we could not compare our results with studies with a similar recruitment design, our data align with the current global systematic synthesised evidence for transgender populations [2,10] and contribute to emerging knowledge about non-binary populations. This is particularly relevant since most European HIV/STI surveillance systems fail to reflect this high likelihood [17-19]. The lack of comprehensive data can result in delayed healthcare access and inadequate public health responses [28]. Consequently, tailored HIV/STI services may not effectively reach these marginalised populations [29]. Our study highlights these gaps, revealing that one in five trans* participants had never tested for HIV, around 15% had never tested for STIs and the majority (70%) had never used PrEP, which is remarkably lower than those observed in MSM populations [30]. These findings indicate substantial gaps in HIV/STI prevention and unmet sexual health needs among trans* individuals, further exacerbating disparities in access to care, and are thus not in line with the European Union’s LGBTIQ Equality Strategy 2020–2025 [31] and the UNAIDS’s End Inequalities initiative [32]. 

Our study, with data from 20 European countries on transgender and non-binary populations, can therefore help to address these data gaps in a pan-European context. Based on our findings, we strongly recommend that future European HIV/STI surveillance and research efforts prioritise the inclusion of transgender and non-binary populations. It is crucial that data collection methods are designed to be comprehensive and appropriate for both groups, ensuring that their experiences are accurately represented, even if sample sizes are relatively small, and as long as that can be safely done in terms of privacy. Data should reflect real-world demographics without excluding or under-reporting these populations [33]. For example, research must be inclusive and respectful of gender identities, using self-reported gender rather than restricting participants to predefined sex or gender categories that may not capture the full diversity of their identities [19].

We also explored other determinants of recent STI diagnoses among both transgender and non-binary individuals. Specifically, our findings reported a higher likelihood of recent STIs among transgender and non-binary individuals living with HIV, using PrEP, engaging in transactional sex or residing in more urban areas, similar to the MSM context [34-36]. This is particularly important given the potential interaction between these factors and the high self-reported prevalence of recent high-risk behaviours among these populations, such as engaging in transactional sex (both receiving and providing) or having unprotected sex (Table 1). In addition, our analysis also identified different determinants/risk factors of recent STI diagnoses between transgender and non-binary individuals, revealing that there are indeed disparities between these two groups. European public health authorities such as the European Centre for Disease Prevention and Control (ECDC) and national public health authorities concerned with prevention of communicable diseases including HIV/STI should be aware of these risk factors, underscoring the importance of prioritising tailored STI screening/prevention services for transgender and non-binary individuals, especially for those with a history of higher-risk behaviours as revealed in this study. Also, our findings provide valuable insights for community-based organisations or non-governmental organisations for HIV/STI or trans* community. Consequently, peer-led interventions can be designed and targeted to further accelerate ending the HIV and other STI epidemics among transgender and non-binary populations.

Furthermore, our study found that non-binary individuals face an even greater likelihood of HIV infection, recent STI diagnoses (particularly syphilis) and a greater variety of recent STIs, compared with transgender individuals. This elevated likelihood likely arises from greater health and well-being challenges, including poorer mental health, limited social support, and lower socioeconomic positions, compared with binary transgender individuals [19,37-40]. Consequently, their disease burdens differ, including HIV and STIs. Another possible reason may be their positionality regarding transactional sex. Compared with transgender individuals, in our samples, non-binary individuals were more likely to receive transactional sex rather than provide transactional sex, indicating that they are more likely to act as clients instead of sex workers in the transactional sex. However, their sexual health literacy, especially on HIV and STI prevention, may differ [41]. While the reported sexual health literacy is high among sex workers [42], the knowledge of HIV/STI prevention may still remain low among their clients [43], resulting in elevated likelihoods of HIV/STIs among non-binary individuals compared with transgender individuals. Therefore, the potential intersectionality of different marginalised identities among transgender and non-binary individuals, such as those who are involved in transactional sex or with other minority backgrounds may require additional attention and tailored HIV/STI service delivery and access.

Our study also has limitations. Firstly, we relied on self-reported survey data without clinically confirmed testing evidence since routine surveillance data for HIV/STIs among transgender and non-binary populations in Europe were unavailable. This may lead to under- or overestimation caused by information biases, self-denial or stigma, and especially in cases of a mismatch between HIV status awareness and HIV testing frequency. However, given that no differences were found between participants with an HIV-unaware and HIV-negative status for any endpoint of our study, we believe this would not significantly bias our results. Secondly, the cross-sectional nature of our data prevents longitudinal assessment, making our findings time-sensitive. Thirdly, the PROTECT survey was designed to understand interest and intention to use a long-acting injectable PrEP modality. Despite this, people with HIV were also included and participated. However, this population may not be as interested in participating in a survey like PROTECT which is geared to understanding the use of HIV prevention strategies for people who do not live with HIV. Consequently, our estimates on the self-reported prevalence of HIV among transgender and non-binary participants in this study may be underestimated. Finally, given the relatively small sample size of transgender/non-binary participants, our study may not be powered sufficiently to investigate the determinants of recent STI diagnoses separately. Consequently, our separate determinantal multivariable logistic regression models for both transgender and non-binary individuals may not uncover all the potential differences in the determinants of recent STI diagnosis. However, given that our study still revealed notable differences between these two groups, our recommendations for a clearer differentiation between transgender and non-binary individuals in European HIV/STI research and surveillance hold their relevance and can be extended to other geographical regions.

## Conclusion

Both transgender and non-binary individuals in Europe are disproportionally affected by HIV and bacterial STIs. Compared with transgender individuals, non-binary individuals face even greater likelihoods of HIV and STIs and show different determinants of recent STI diagnosis. There should be a priority set for recognising the sexual healthcare needs of both communities and, subsequently, for a clearer differentiation between transgender and non-binary individuals in HIV/STI research and surveillance efforts. Thus, provided data could ensure that the different needs of marginalised populations are not overlooked. In turn, we can improve tailored HIV/STI services, enhancing HIV/STI control in Europe, and help reaching the HIV-related Sustainable Development Goals (SDGs) set by the United Nations. It is vital that transgender and non-binary individuals continue to be included in existing HIV/STI services. Monitoring on a European level and national-level interventions should prioritise reaching these populations. The time is now to commence delivering targeted prevention strategies, such as HIV/STI testing, PrEP and doxycycline post-exposure prophylaxis for bacterial STI prevention (DoxyPEP), to high-risk individuals identified in this study.

## *Positionality statement

Two of our authors speak from a first-person perspective of the trans* community to ensure appropriate classifications, definitions and use of language, as well as analytical approaches.

## Supplementary Data

500000 Supplement
