# Supplementary material for: Likelihood of HIV and recent bacterial sexually transmitted infections among transgender and non-binary individuals in 20 European countries, October 2023 to April 2024
Source: Euro Surveill. 2024 Nov 28;29(48):2400347. doi: 10.2807/1560-7917.ES.2024.29.48.2400347 (PMC11605802; doi:10.2807/1560-7917.ES.2024.29.48.2400347)
Supplement: Supplement [file 24-00347_WANG_Supplement.pdf]

Supplementary materials

"This supplementary material is hosted by *Eurosurveillance* as supporting information alongside the article [Likelihood of HIV and Recent Bacterial Sexually Transmitted Infections among Transgender and Non-binary Individuals in 20 European Countries, October 2023 – April 2024], on behalf of the authors, who remain responsible for the accuracy and appropriateness of the content. The same standards for ethics, copyright, attributions and permissions as for the article apply. Supplements are not edited by *Eurosurveillance* and the journal is not responsible for the maintenance of any links or email addresses provided therein."

Table S1. Prevalence of self-reported HIV status and recent STI diagnoses among transgender and non-binary individuals in 20 European countries, October 2023–April 2024 (n = 452)

	Self-reported status	Transgender (n=178)		Non-binary (n=274)		Total (n=452)	
		n	%	n	%	n	%
**HIV**	**Unware**	27	15.2	36	13.1	63	13.9
	**Negative**	146	82.0	223	81.4	369	81.6
	**Positive**	5	2.8	15	5.5	20	4.4
**Syphilis in the preceding 6 month**	**Yes**	12	6.7	41	15.0	53	11.7
	**No**	166	93.3	233	85.0	399	88.3
**Gonorrhoea in the preceding 6 month**	**Yes**	28	15.7	70	25.5	98	21.7
	**No**	150	84.3	204	74.5	354	78.3
**Chlamaydia in the preceding 6 month**	**Yes**	35	19.7	57	20.8	92	20.4
	**No**	143	80.3	217	79.2	360	79.6
**Number of recent STIs**	**0**	128	71.9	165	60.2	293	64.8
	**1**	30	16.9	65	23.7	95	21.0
	**2**	15	8.4	29	10.6	44	9.7
	**3**	5	2.8	15	5.5	20	4.4

Table S2 Univariable logistic regression on the likelihood of recent self-reported syphilis, gonorrhoea, and chlamydia diagnosis in the preceding six months among HIV-negative transgender and non-binary individuals who ever tested for STIs, in 20 European countries, October 2023 – April 2024

Variable		Recent syphilis diagnosis				Recent gonorrhoea diagnosis				Recent chlamydia diagnosis			
		OR	Lower CI	Upper CI	p value	OR	Lower CI	Upper CI	p value	OR	Lower CI	Upper CI	p value
Gender group	Transgender	Ref	-	-	-	Ref	-	-	-	Ref	-	-	-
	non-binary	2,43	1,28	4,97	0,010	1,84	1,14	3,03	0,014	1,07	0,67	1,73	0,769
HiV status	Negative	Ref	-	-	-	Ref	-	-	-	Ref	-	-	-
	Unaware	0,75	0,25	1,83	0,564	0,44	0,18	0,94	0,049	0,38	0,14	0,85	0,031
	Positive	8,71	3,37	22,57	0,000	2,86	1,12	7,15	0,024	1,55	0,53	3,99	0,386
Age	18-24	Ref	-	-	-	Ref	-	-	-	Ref	-	-	-
	25-29	2,28	0,81	7,39	0,135	2,59	1,20	5,95	0,018	3,14	1,39	7,80	0,009
	30-39	2,78	1,05	8,71	0,053	3,60	1,75	8,03	0,001	4,10	1,89	9,94	0,001
	40-49	5,64	2,05	18,17	0,002	2,44	1,02	6,05	0,047	2,84	1,12	7,61	0,030
	50-59	1,57	0,22	7,77	0,605	2,67	0,83	8,09	0,086	3,41	1,02	10,94	0,039
	60-69	0,00	0,00	Inf	0,986	2,22	0,31	10,52	0,351	2,84	0,39	13,99	0,231
	70+	5,37	0,69	30,60	0,069	4,45	0,84	19,90	0,056	9,10	1,94	41,86	0,004
Education	Below secondary education	Ref	-	-	-	Ref	-	-	-	Ref	-	-	-
	Secondary education (high school or equivalent)	0,89	0,27	4,03	0,865	0,34	0,13	0,96	0,032	1,32	0,42	5,88	0,667
	Bachelor degree (university or equivalent)	1,04	0,31	4,72	0,954	0,89	0,35	2,44	0,805	2,03	0,64	8,97	0,277
	Master degree (university or equivalent)	1,18	0,35	5,42	0,808	1,14	0,45	3,16	0,794	3,36	1,07	14,88	0,062
	PhD / Doctorate	1,10	0,18	6,49	0,917	1,63	0,49	5,56	0,425	2,02	0,44	10,88	0,376
Employment	Employed	Ref	-	-	-	Ref	-	-	-	Ref	-	-	-
	Other	0,37	0,09	1,09	0,111	0,42	0,16	0,92	0,043	0,39	0,14	0,90	0,041
	Retired/Medical leave	1,00	0,32	2,57	0,997	0,55	0,20	1,30	0,204	0,74	0,29	1,71	0,508
	Student	0,44	0,16	1,02	0,076	0,34	0,16	0,67	0,003	0,38	0,18	0,76	0,009
	Unemployed	0,66	0,24	1,55	0,369	0,65	0,31	1,28	0,230	0,66	0,31	1,31	0,251
Perceived income	Living really comfortably on present income	Ref	-	-	-	Ref	-	-	-	Ref	-	-	-
	Living comfortably on present income	5,00	0,62	31,02	0,091	2,68	0,61	11,27	0,174	1,45	0,28	6,08	0,625
	Neither comfortable nor struggling on present income	2,19	0,79	7,76	0,169	0,93	0,49	1,81	0,817	0,77	0,41	1,48	0,427
	Struggling on present income	2,66	0,91	9,71	0,096	0,88	0,43	1,82	0,722	0,55	0,26	1,15	0,114
	Really struggling on present income	3,14	1,07	11,48	0,053	0,82	0,38	1,73	0,593	0,66	0,31	1,38	0,265
Migration background	Non migrant	Ref	-	-	-	Ref	-	-	-	Ref	-	-	-
	First Generation migrant	1,94	1,05	3,56	0,032	1,05	0,63	1,72	0,855	1,04	0,62	1,73	0,875
	Second Generation migrant	0,84	0,24	2,28	0,758	0,71	0,29	1,52	0,404	0,77	0,32	1,66	0,533

Place of residence	A very big city or town (a million or more people)	Ref	-	-	-	Ref	-	-	-	Ref	-	-	-
	A big city or town (500,000-999,999 people)	0,40	0,14	0,97	0,058	0,61	0,31	1,14	0,129	0,58	0,30	1,11	0,108
	A medium-sized city or town (100,000-499,999 people)	0,71	0,35	1,41	0,330	0,39	0,21	0,70	0,002	0,35	0,19	0,65	0,001
	A small city or town (10,000-99,999 people)	0,37	0,13	0,91	0,041	0,41	0,20	0,79	0,010	0,29	0,13	0,59	0,001
	A village / the countryside (less than 10,000 people)	0,30	0,07	0,91	0,057	0,17	0,05	0,45	0,001	0,28	0,10	0,66	0,007
Relationship status	Single	Ref	-	-	-	Ref	-	-	-	Ref	-	-	-
	Dating	1,46	0,72	3,03	0,295	1,35	0,75	2,43	0,317	1,71	0,92	3,22	0,094
	In a monogamous Relationship	0,69	0,25	1,71	0,437	0,72	0,34	1,48	0,383	0,49	0,19	1,17	0,128
	In an open/polyamorous relationship	0,80	0,33	1,84	0,602	1,30	0,70	2,42	0,404	2,60	1,40	4,93	0,003
HIV testing frequency	Frequently testing	Ref	-	-	-	Ref	-	-	-	Ref	-	-	-
	Every six months	1,25	0,60	2,59	0,544	1,07	0,61	1,87	0,822	0,76	0,42	1,35	0,357
	Less than once per year	0,28	0,04	1,03	0,099	0,20	0,06	0,54	0,004	0,19	0,06	0,52	0,003
	Never	0,00	0,00	Inf	0,987	0,00	0,00	287,19	0,979	0,02	0,00	0,10	0,000
	Once per year	0,55	0,19	1,37	0,224	0,29	0,13	0,61	0,002	0,32	0,14	0,65	0,003
Condomless anal intercourse in the preceding six months	No	Ref	-	-	-	Ref	-	-	-	Ref	-	-	-
	Yes	1,61	0,71	4,33	0,294	1,71	0,90	3,56	0,123	1,39	0,74	2,82	0,335
Unprotected sex	Yes	Ref	-	-	-	Ref	-	-	-	Ref	-	-	-
	No	1,33	0,72	2,40	0,352	2,04	1,28	3,25	0,003	2,21	1,37	3,55	0,001
Chemsex in the preceding six months	No	Ref	-	-	-	Ref	-	-	-	Ref	-	-	-
	Yes	4,26	2,19	8,10	0,000	2,34	1,29	4,15	0,004	2,82	1,57	5,02	0,000
Number of sex partners in the preceding six month	0	Ref	-	-	-	Ref	-	-	-	Ref	-	-	-
	1	0,00	0,00	Inf	0,989	1,50	0,18	30,89	0,729	0,24	0,01	2,55	0,246
	2-10	0,00	0,00	Inf	0,988	8,88	1,80	160,76	0,035	5,25	1,50	33,30	0,027
	11-50	0,00	0,00	Inf	0,987	26,52	5,41	479,79	0,002	10,52	2,98	67,03	0,002
	51-100	0,00	0,00	Inf	0,987	17,08	2,59	339,07	0,012	3,38	0,51	27,56	0,206
	101-150	0,00	0,00	Inf	0,987	18,50	2,01	411,19	0,018	9,00	1,25	80,58	0,030
	150+	0,00	0,00	Inf	0,987	11,38	2,09	212,54	0,022	4,39	1,07	29,81	0,067
Transactional sex (receiving) in the preceding 6 months	No	Ref	-	-	-	Ref	-	-	-	Ref	-	-	-
	Yes	4,89	2,56	9,22	0,000	1,51	0,81	2,70	0,179	2,38	1,32	4,21	0,003
Transactional sex (providing) in the preceding 6 months	No	Ref	-	-	-	Ref	-	-	-	Ref	-	-	-
	Yes	2,13	1,18	3,80	0,011	0,94	0,57	1,52	0,805	1,43	0,88	2,30	0,140

Oral PrEP use status	Current	Ref	-	-	-	Ref	-	-	-	Ref	-	-	-
	Former	1,00	0,36	2,51	0,995	0,63	0,27	1,41	0,267	0,73	0,31	1,63	0,451
	Naive	0,15	0,07	0,32	0,000	0,13	0,07	0,22	0,000	0,15	0,09	0,26	0,000

Table S3 Univariable logistic regression on the likelihood of recent self-reported syphilis, gonorrhoea, and chlamydia diagnosis in the preceding six months among HIV-negative transgender individuals who ever tested for STIs, in 20 European countries, October 2023 – April 2024

Variable		Recent syphilis diagnosis				Recent gonorrhoea diagnosis				Recent chlamydia diagnosis			
		OR	Lower CI	Upper CI	p value	OR	Lower CI	Upper CI	p value	OR	Lower CI	Upper CI	p value
HiV status	Negative	Ref	-	-	-	Ref	-	-	-	Ref	-	-	-
	Unaware	0,59	0,03	3,32	0,619	0,64	0,14	2,01	0,487	0,29	0,04	1,03	0,099
	Positive	10,15	1,22	69,35	0,018	1,27	0,06	9,07	0,833	0,89	0,04	6,29	0,919
Age	18-24	Ref	-	-	-	Ref	-	-	-	Ref	-	-	-
	25-29	1,73	0,16	38,14	0,658	2,16	0,56	10,59	0,290	11,92	2,16	223,28	0,021
	30-39	3,25	0,46	64,97	0,301	4,11	1,21	18,98	0,038	11,70	2,15	218,22	0,021
	40-49	7,43	1,02	150,41	0,081	1,68	0,29	9,79	0,545	9,75	1,45	193,64	0,044
	50-59	4,88	0,18	132,39	0,280	1,54	0,07	13,96	0,722	19,50	2,12	433,14	0,016
	60-69	0,00	-Inf	Inf	0,996	0,00	-Inf	Inf	0,990	39,00	1,06	1983,12	0,035
	70+	0,00	-Inf	Inf	0,995	6,17	0,25	87,85	0,182	78,00	4,40	3423,86	0,006
Education	Below secondary education	Ref	-	-	-	Ref	-	-	-	Ref	-	-	-
	Secondary education (high school or equivalent)	0,15	0,02	1,40	0,076	0,58	0,12	4,25	0,533	1,90	0,32	36,59	0,556
	Bachelor degree (university or equivalent)	0,23	0,03	2,08	0,162	1,05	0,22	7,60	0,957	3,00	0,50	57,88	0,318
	Master degree (university or equivalent)	0,69	0,12	5,45	0,688	1,83	0,39	13,31	0,481	6,78	1,13	130,68	0,081
	PhD / Doctorate	2,75	0,26	30,43	0,382	2,75	0,26	30,43	0,382	2,40	0,08	69,43	0,562
Employment	Employed	Ref	-	-	-	Ref	-	-	-	Ref	-	-	-
	Other	0,33	0,02	1,83	0,296	0,53	0,12	1,76	0,344	0,41	0,09	1,33	0,178
	Retired/Medical leave	0,49	0,03	2,83	0,508	0,83	0,18	2,90	0,791	0,64	0,14	2,20	0,517
	Student	0,00	-Inf	Inf	0,994	0,35	0,05	1,36	0,185	0,27	0,04	1,03	0,095
	Unemployed	0,00	-Inf	Inf	0,994	0,35	0,05	1,36	0,185	0,79	0,24	2,24	0,673
Perceived income	Living really comfortably on present income	Ref	-	-	-	Ref	-	-	-	Ref	-	-	-
	Living comfortably on present income	0,00	0,00	NA	0,992	0,00	0,00	Inf	0,987	2,40	0,09	64,84	0,550
	Neither comfortable nor struggling on present income	0,00	0,00	NA	0,993	0,84	0,27	2,73	0,763	0,68	0,26	1,80	0,428
	Struggling on present income	0,00	0,00	NA	0,993	0,57	0,13	2,18	0,412	0,46	0,14	1,43	0,189
	Really struggling on present income	0,00	0,00	NA	0,993	0,84	0,25	2,86	0,771	0,29	0,08	0,92	0,042
Migration background	Non migrant	Ref	-	-	-	Ref	-	-	-	Ref	-	-	-
	First Generation migrant	7,18	1,97	34,05	0,005	1,81	0,72	4,43	0,193	0,80	0,31	1,90	0,628
	Second Generation migrant	1,59	0,08	13,10	0,694	1,41	0,37	4,46	0,577	1,09	0,33	3,08	0,883
Place of residence	A very big city or town (a million or more people)	Ref	-	-	-	Ref	-	-	-	Ref	-	-	-

	A big city or town (500,000-999,999 people)	0,94	0,18	4,59	0,942	1,02	0,34	2,94	0,978	0,90	0,31	2,54	0,836
	A medium-sized city or town (100,000-499,999 people)	1,11	0,27	4,75	0,885	0,46	0,14	1,37	0,171	0,57	0,20	1,57	0,277
	A small city or town (10,000-99,999 people)	0,00	-Inf	Inf	0,993	0,44	0,11	1,47	0,202	0,28	0,06	1,00	0,069
	A village / the countryside (less than 10,000 people)	0,00	-Inf	Inf	0,995	0,00	0,00	Inf	0,991	1,12	0,30	3,76	0,859
Relationship status	Single	Ref	-	-	-	Ref	-	-	-	Ref	-	-	-
	Dating	3,03	0,47	59,31	0,320	1,62	0,55	5,42	0,399	2,72	0,91	10,13	0,096
	In a monogamous Relationship	4,77	0,66	96,02	0,172	1,37	0,37	5,19	0,635	0,80	0,15	3,88	0,777
	In an open/polyamorous relationship	2,00	0,18	44,14	0,578	0,75	0,17	3,09	0,693	3,78	1,17	14,74	0,036
HIV testing frequency	Frequently testing	Ref	-	-	-	Ref	-	-	-	Ref	-	-	-
	Every six months	0,00	0,00	Inf	0,993	2,00	0,64	8,79	0,283	2,71	0,89	11,84	0,118
	Less than once per year	0,75	0,16	2,64	0,678	1,11	0,45	2,57	0,821	1,25	0,56	2,72	0,571
	Never	4,11	1,02	14,51	0,033	2,28	0,75	6,24	0,120	2,73	1,00	7,05	0,041
	Once per year	1,00	0,00	Inf	1,000	1,00	0,00	Inf	1,000	1,00	0,00	Inf	1,000
Condomless anal intercourse in the preceding six months	No	Ref	-	-	-	Ref	-	-	-	Ref	-	-	-
	Yes	1,00	0,00	Inf	1,000	0,00	0,00	Inf	0,993	0,00	0,00	Inf	0,993
Unprotected sex	Yes	Ref	-	-	-	Ref	-	-	-	Ref	-	-	-
	No	0,00	0,00	NA	0,996	0,00	0,00	Inf	0,993	0,00	0,00	Inf	0,993
Chemsex in the preceding six months	No	Ref	-	-	-	Ref	-	-	-	Ref	-	-	-
	Yes	0,00	0,00	NA	0,996	0,00	0,00	Inf	0,993	0,00	0,00	Inf	0,993
Number of sex partners in the preceding six month	0	Ref	-	-	-	Ref	-	-	-	Ref	-	-	-
	1	0,00	0,00	NA	0,996	0,00	0,00	Inf	0,993	0,00	0,00	Inf	0,993
	2-10	0,00	-Inf	Inf	0,992	0,00	0,00	Inf	0,987	0,00	0,00	Inf	0,987
	11-50	0,49	0,07	2,28	0,400	1,00	0,37	2,64	0,995	0,75	0,29	1,86	0,535
	51-100	0,29	0,01	1,82	0,264	0,21	0,03	0,85	0,053	0,30	0,08	0,91	0,048
	101-150	0,00	0,00	NA	0,995	0,00	0,00	Inf	0,993	0,00	0,00	Inf	0,993
	150+	0,34	0,02	2,16	0,332	0,25	0,04	1,02	0,087	0,08	0,00	0,42	0,016
Transactional sex (receiving) in the preceding 6 months	No	Ref	-	-	-	Ref	-	-	-	Ref	-	-	-
	Yes	5,43	1,32	19,69	0,012	1,08	0,24	3,58	0,908	1,19	0,32	3,59	0,773
Transactional sex (providing) in the preceding 6 months	No	Ref	-	-	-	Ref	-	-	-	Ref	-	-	-
	Yes	2,68	0,82	9,40	0,105	0,99	0,41	2,26	0,977	1,24	0,57	2,63	0,578
Oral PrEP use status	Current	Ref	-	-	-	Ref	-	-	-	Ref	-	-	-
	Former	2,13	0,26	13,08	0,428	0,93	0,18	4,01	0,924	0,48	0,09	2,00	0,331
	Naive	0,28	0,06	1,24	0,083	0,23	0,09	0,57	0,001	0,13	0,05	0,30	0,000

Table S4 Univariable logistic regression on the likelihood of recent self-reported syphilis, gonorrhoea, and chlamydia diagnosis in the preceding six months among HIV-negative non-binary individuals who ever tested for STIs, in 20 European countries, October 2023 – April 2024

Variable		Recent syphilis diagnosis				Recent gonorrhoea diagnosis				Recent chlamydia diagnosis			
		OR	Lower CI	Upper CI	p value	OR	Lower CI	Upper CI	p value	OR	Lower CI	Upper CI	p value
HiV status	Negative	Ref	-	-	-	Ref	-	-	-	Ref	-	-	-
	Unaware	0,84	0,24	2,31	0,752	0,36	0,10	0,94	0,061	0,46	0,13	1,22	0,157
	Positive	7,65	2,56	23,37	0,000	3,25	1,12	9,66	0,029	1,82	0,55	5,39	0,293
Age	18-24	Ref	-	-	-	Ref	-	-	-	Ref	-	-	-
	25-29	2,55	0,80	9,74	0,133	2,91	1,15	8,05	0,029	1,89	0,72	5,40	0,209
	30-39	2,67	0,89	9,89	0,102	3,38	1,41	9,07	0,010	3,00	1,24	8,08	0,020
	40-49	5,22	1,63	20,17	0,008	2,82	1,00	8,42	0,053	1,86	0,61	5,77	0,273
	50-59	0,86	0,04	6,34	0,895	3,10	0,80	11,49	0,090	1,59	0,31	6,58	0,537
	60-69	0,00	0,00	Inf	0,987	2,48	0,32	13,50	0,319	1,06	0,05	7,38	0,959
	70+	6,88	0,79	49,52	0,056	3,71	0,46	23,16	0,169	3,71	0,46	23,16	0,169
Education	Below secondary education	Ref	-	-	-	Ref	-	-	-	Ref	-	-	-
	Secondary education (high school or equivalent)	2,40	0,42	45,36	0,417	0,22	0,06	0,85	0,022	1,01	0,24	7,00	0,987
	Bachelor degree (university or equivalent)	2,40	0,42	45,48	0,418	0,68	0,21	2,43	0,529	1,50	0,36	10,26	0,618
	Master degree (university or equivalent)	2,03	0,34	39,09	0,517	0,75	0,22	2,72	0,644	2,09	0,50	14,32	0,365
	PhD / Doctorate	0,71	0,03	19,04	0,812	1,02	0,23	4,57	0,981	1,57	0,26	12,88	0,636
Employment	Employed	Ref	-	-	-	Ref	-	-	-	Ref	-	-	-
	Other	0,43	0,07	1,59	0,271	0,39	0,11	1,09	0,097	0,38	0,09	1,18	0,133
	Retired/Medical leave	1,51	0,40	4,73	0,502	0,43	0,10	1,41	0,206	0,85	0,23	2,60	0,795
	Student	0,54	0,19	1,32	0,200	0,31	0,13	0,67	0,005	0,42	0,17	0,93	0,041
	Unemployed	1,02	0,35	2,60	0,974	0,81	0,34	1,79	0,610	0,57	0,20	1,42	0,257
Perceived income	Living really comfortably on present income	Ref	-	-	-	Ref	-	-	-	Ref	-	-	-
	Living comfortably on present income	1,50	0,07	12,60	0,736	1,05	0,14	5,74	0,953	1,38	0,18	7,69	0,727
	Neither comfortable nor struggling on present income	1,29	0,43	4,76	0,675	0,88	0,40	2,04	0,756	0,89	0,38	2,22	0,794
	Struggling on present income	1,89	0,61	7,19	0,299	0,93	0,39	2,29	0,872	0,65	0,24	1,78	0,396
	Really struggling on present income	2,50	0,76	9,79	0,150	0,83	0,31	2,20	0,704	1,22	0,45	3,35	0,700
Migration background	Non migrant	Ref	-	-	-	Ref	-	-	-	Ref	-	-	-
	First Generation migrant	1,28	0,61	2,61	0,500	0,81	0,43	1,48	0,498	1,19	0,62	2,23	0,587
	Second Generation migrant	0,82	0,19	2,62	0,767	0,49	0,14	1,38	0,218	0,52	0,12	1,62	0,313
Place of residence	A very big city or town (a million or more people)	Ref	-	-	-	Ref	-	-	-	Ref	-	-	-

	A big city or town (500,000-999,999 people)	0,27	0,06	0,86	0,045	0,48	0,20	1,08	0,085	0,46	0,19	1,07	0,081
	A medium-sized city or town (100,000-499,999 people)	0,63	0,27	1,43	0,276	0,37	0,17	0,76	0,008	0,27	0,12	0,60	0,002
	A small city or town (10,000-99,999 people)	0,51	0,17	1,34	0,192	0,41	0,17	0,92	0,037	0,30	0,11	0,71	0,010
	A village / the countryside (less than 10,000 people)	0,36	0,08	1,18	0,125	0,22	0,06	0,62	0,009	0,05	0,00	0,28	0,005
Relationship status	Single	Ref	-	-	-	Ref	-	-	-	Ref	-	-	-
	Dating	1,82	0,82	4,10	0,143	1,50	0,73	3,09	0,266	1,43	0,64	3,15	0,379
	In a monogamous Relationship	0,36	0,08	1,16	0,119	0,55	0,20	1,35	0,211	0,41	0,11	1,21	0,134
	In an open/polyamorous relationship	0,76	0,29	1,88	0,555	1,65	0,82	3,36	0,162	2,32	1,11	4,94	0,026
HIV testing frequency	Frequently testing	Ref	-	-	-	Ref	-	-	-	Ref	-	-	-
	Every six months	1,07	0,44	2,98	0,894	1,55	0,71	3,76	0,297	0,96	0,44	2,25	0,914
	Less than once per year	1,59	0,78	3,14	0,189	2,75	1,56	4,86	0,000	3,13	1,71	5,74	0,000
	Never	4,31	1,97	9,25	0,000	2,33	1,14	4,71	0,019	2,87	1,37	5,90	0,004
	Once per year	0,00	0,00	Inf	0,991	1,81	0,22	37,72	0,614	0,28	0,01	3,04	0,305
Condomless anal intercourse in the preceding six months	No	Ref	-	-	-	Ref	-	-	-	Ref	-	-	-
	Yes	0,00	0,00	Inf	0,988	26,00	1,80	750,38	0,022	12,50	1,07	175,89	0,042
Unprotected sex	Yes	Ref	-	-	-	Ref	-	-	-	Ref	-	-	-
	No	0,00	0,00	Inf	0,989	25,21	4,89	463,29	0,002	6,85	1,81	44,94	0,014
Chemsex in the preceding six months	No	Ref	-	-	-	Ref	-	-	-	Ref	-	-	-
	Yes	0,00	0,00	Inf	0,989	9,45	1,56	182,50	0,041	3,80	0,82	27,33	0,117
Number of sex partners in the preceding six month	0	Ref	-	-	-	Ref	-	-	-	Ref	-	-	-
	1	0,00	0,00	Inf	0,989	7,26	1,39	133,80	0,059	3,73	0,99	24,44	0,090
	2-10	0,00	0,00	Inf	0,990	0,00	0,00	Inf	0,983	0,04	0,00	0,17	0,001
	11-50	1,62	0,69	3,80	0,266	1,07	0,53	2,15	0,842	0,78	0,36	1,62	0,505
	51-100	0,67	0,20	1,92	0,475	0,32	0,12	0,77	0,015	0,33	0,11	0,84	0,027
	101-150	0,00	0,00	Inf	0,989	13,00	1,81	266,11	0,027	1,92	0,21	17,58	0,536
	150+	0,27	0,01	1,48	0,217	0,18	0,03	0,69	0,029	0,36	0,08	1,19	0,127
Transactional sex (receiving) in the preceding 6 months	No	Ref	-	-	-	Ref	-	-	-	Ref	-	-	-
	Yes	4,33	2,06	9,02	0,000	1,52	0,75	2,99	0,231	3,12	1,55	6,16	0,001
Transactional sex (providing) in the preceding 6 months	No	Ref	-	-	-	Ref	-	-	-	Ref	-	-	-
	Yes	2,21	1,11	4,36	0,023	0,98	0,53	1,77	0,955	1,60	0,85	2,95	0,136
Oral PrEP use status	Current	Ref	-	-	-	Ref	-	-	-	Ref	-	-	-
	Former	0,76	0,23	2,24	0,640	0,51	0,18	1,34	0,182	0,90	0,32	2,38	0,831
	Naive	0,13	0,05	0,31	0,000	0,10	0,05	0,19	0,000	0,17	0,08	0,34	0,000
